# PlasmidScope: a comprehensive plasmid database with rich annotations and online analytical tools

**DOI:** 10.1093/nar/gkae930

**Published:** 2024-10-23

**Authors:** Yinhu Li, Xikang Feng, Xuhua Chen, Shuo Yang, Zicheng Zhao, Yu Chen, Shuai Cheng Li

**Affiliations:** CAS Key Laboratory of Brain Connectome and Manipulation, the Brain Cognition and Brain Disease Institute, Shenzhen Institute of Advanced Technology, Chinese Academy of Sciences, Shenzhen 518055, China; SIAT-HKUST Joint Laboratory for Brain Science, Chinese Academy of Sciences, Shenzhen 518055, China; Shenzhen-Hong Kong Institute of Brain Science, Shenzhen Fundamental Research Institutions, Shenzhen 518055, China; School of Software, Northwestern Polytechnical University, Xi’an 710072, China; Research & Development Institute, Northwestern Polytechnical University, Shenzhen 518063, China; SIAT-HKUST Joint Laboratory for Brain Science, Chinese Academy of Sciences, Shenzhen 518055, China; Department of Computer Science, City University of Hong Kong, Hong Kong, China; OmicLab Limited, Science Park East Avenue, Hong Kong Science Park, Hong Kong, China; CAS Key Laboratory of Brain Connectome and Manipulation, the Brain Cognition and Brain Disease Institute, Shenzhen Institute of Advanced Technology, Chinese Academy of Sciences, Shenzhen 518055, China; SIAT-HKUST Joint Laboratory for Brain Science, Chinese Academy of Sciences, Shenzhen 518055, China; Shenzhen-Hong Kong Institute of Brain Science, Shenzhen Fundamental Research Institutions, Shenzhen 518055, China; Department of Computer Science, City University of Hong Kong, Hong Kong, China; City University of Hong Kong Shenzhen Research Institute, Shenzhen 518057, China

## Abstract

Plasmids are extrachromosomal genetic molecules that replicate independent of chromosomes in bacteria, archaea, and eukaryotic organisms. They contain diverse functional elements and are capable of horizontal gene transfer among hosts. While existing plasmid databases have archived plasmid sequences isolated from individual microorganisms or natural environments, there is a need for a comprehensive, standardized, and annotated plasmid database to address the vast accumulation of plasmid sequences. Here, we propose PlasmidScope (https://plasmid.deepomics.org/), a plasmid database offering comprehensive annotations, automated online analysis, and interactive visualization. PlasmidScope harbors a substantial collection of 852 600 plasmids curated from 10 repositories. Along with consolidated background information, PlasmidScope utilizes 12 state-of-the-art tools and provides comprehensive annotations for the curated plasmids, covering genome completeness, topological structure, mobility, host source, tRNA, tmRNA, signal peptides, transmembrane proteins and CRISPR/Cas systems. PlasmidScope offers diverse functional annotations for its 25 231 059 predicted genes from 9 databases as well as corresponding protein structures predicted by ESMFold. In addition, PlasmidScope integrates online analytical modules and interactive visualization, empowering researchers to delve into the complexities of plasmids.

## Graphical Abstract

**Graphical Abstract:**
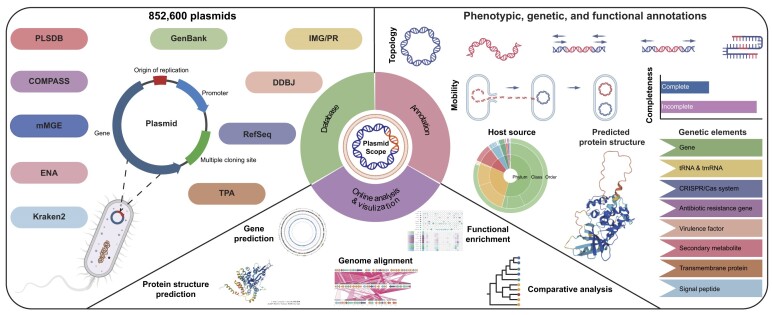

## Introduction

A plasmid is an extrachromosomal genetic molecule that replicates independent of chromosomes in bacteria, archaea, and eukaryotic organisms. They carry diverse functional elements and facilitates horizontal gene transfer (1,2). As a class of mobile genetic elements, plasmids have diverse functional elements, such as antibiotic resistance genes (ARGs) (3), virulence factors (VFs) (4), and metabolic compounds (5). They also contribute to genetic diversity (6,7) and rapid adaptation of the host to various environments (8). For example, ARGs in plasmids enable the survival of pathogens under antibiotic exposure, leading to antibiotic-resistant infections, which is a serious challenge in clinical settings (8–10). In addition, the horizontal gene transfer capabilities of plasmids, including conjugation, transformation, and transduction, enhance the spread of genetic elements across different microorganisms and environments (11), which might accelerate host evolution by providing highly diverse and multicopy plasmid-encoded genes. Therefore, plasmids play crucial roles in evolutionary dynamics and ecological diversity.

The physiological features of plasmids, such as topology and mobility, as well as their genetic elements, including genes, signal peptides, and CRISPR/Cas systems, provide a foundation for understanding horizontal gene transfer, evolutionary dynamics, and ecological diversity (12–16). Topology and mobility are key features of the horizontal gene transfer capability of plasmids: topology influences plasmid stability and replication within hosts (12,17), while mobility determines whether plasmids can spread among different microorganisms through conjugation, transformation, or transduction (12,13). Plasmid-encoded genes are the basic genetic materials spread in different microbial populations and promote evolutionary dynamics (14). Plasmid-encoded signal peptides direct proteins to the cell membrane or secretion systems, facilitating signal transmission and promoting ecological diversity (18). In addition, plasmid-carried CRISPR/Cas systems enable the host to acquire immune memory, allowing the quick adaption of the hosts to viral or foreign DNA threats (19). Thus, the physiological and genetic features of plasmids are crucial for understanding plasmid structures and have bioengineering or clinical applications.

Several plasmid databases that organize the physiological and genetic features of plasmids have been developed in recent years. Plasmid databases, such as PLSDB (20), COMPASS (21) and pATLAS (22), retrieved reliable plasmids from GenBank or the RefSeq database (23,24), providing genetic characteristics and host information as well as supporting web-based data manipulation to assist plasmid-related queries and identification. However, these plasmid databases are typically limited to single microorganisms and therefore do not capture the diversity of plasmids in natural environments (25). With the immense accumulation of next-generation sequencing data and the rapid development of plasmid identification algorithms, some plasmid databases, such as IMG/PR (25) and mMGE (26), have expanded the identification of plasmids based on assembled metagenomes and metatranscriptomes, boosting the number of cataloged plasmids from tens of thousands to millions. Yet, these plasmid databases seldom provide information about the structures of proteins or genetic elements, such as signal peptides, transmembrane proteins, and CRISPR/Cas systems, nor do they facilitate cross-comparison or querying among different databases.

To this end, we propose PlasmidScope (https://plasmid.deepomics.org/), a comprehensive plasmid database offering rich annotations, interactive visualization, and online analysis of curated plasmids. PlasmidScope contains an extensive collection of over 1 million plasmids curated from 10 public repositories. PlasmidScope also provides detailed, downloadable annotations for its curated plasmids, including topological annotation, mobility prediction, completeness assessment, host taxonomy, functional annotations, protein structure prediction, signal peptide and transmembrane protein prediction and CRISPR/Cas system prediction. In addition, PlasmidScope supports interactive visualization of its curated database and related annotation results and contains online analytical modules for custom plasmid analysis.

## Materials and methods

### Plasmid collection and curation

To amass a comprehensive collection of plasmid sequences, we performed an extensive search across public repositories, including GenBank (23), RefSeq (24), ENA (27), DDBJ (28), Kraken2 (29) and TPA (30), focusing on the keyword ‘plasmid’. We searched ‘plasmid’ under the Nucleotide category on the NCBI website. Given that the GenBank, ENA and DDBJ databases are synchronized through the INSDC initiative, we selected the options for ‘GenBank’, ‘DDBJ’, ‘ENA’, ‘RefSeq’, ‘RefSeq’, ‘PDB’ and ‘TPA’ under the ‘Source Databases’ section on the search page. This allowed us to download plasmids while differentiating by their source database. We also included plasmids gleaned from published datasets, such as PLSDB (20), COMPASS (21), IMG/PR (25) and mMGE (26) (Supplementary Table S1). Within these datasets, our collection extended beyond plasmid sequences to include host taxonomic data, topological structures, and assessment of their completeness.

Following plasmid data collection, we curated the data as follows: First, we filtered out plasmids with a genomic length less than 200 bp, GC content less than 10%, or GC content >90%. Second, we removed the duplicate plasmids with identical plasmid sequences within each database. Third, we identified the non-redundant plasmids across the 10 plasmid databases (Supplementary Figure S1). To identify the duplicated plasmids, we applied MMseqs2 (version 15.6f452) to detect plasmids with identical sequences (clustering parameters: ’*–cov-mode 0 -c 1.0 –min-seq-id 1.0*’) (31). We retained the deduplicated plasmids as the final curated plasmid database and included them in the “All” table in the ‘Plasmid list’ page on the website of PlasmidScope. For the redundant plasmids with multiple entries, we consolidated them into the same row of the “All” table and provided links to their source databases. To enhance the connectivity between PlasmidScope and the source databases, we also provide other tables tagged with ‘PLSDB’, ‘IMG/PR’, ‘COMPASS’, ‘ENA’, ‘GenBank’, etc., containing original plasmids from the specified databases.

### Host taxonomic assignment

We collected the host taxonomic data from each dataset. Given that the above datasets were released at different times and that the taxonomic information might vary by version, we performed rigorous standardization. Accordingly, we aligned the host taxonomic information across all datasets to conform to the most current version available from the NCBI (i.e. 202406) (32). This ensures a unified and up-to-date reference point for the host species associated with each plasmid, facilitating the accuracy and consistency of information.

### Plasmid clustering and mobility prediction

To identify plasmid cluster assignments, we used the MOB-typer tool from MOB-suite (v3.1.8) (33) using the default parameters and databases. To obtain higher clustering resolution, we provided cluster and subcluster information for the plasmids after MOB-typing analysis. We applied Mash embedded in MOB-suite for plasmid clustering based on the fast genomic distance estimation. This strategy leverages the genomic sequence of the plasmids for analysis, transcending the limitations of relying on specific biomarkers. MOB-typer also predicts the mobility of plasmids as conjugative, mobilizable, or non-mobilizable (33).

### Annotation of plasmid genes and genomic elements

We performed gene annotation for the plasmid genomes using Prokka (v1.11) (34), which integrates Prodigal (v2.6.3) (35), to identify the open reading frames, along with ARAGORN (v1.2.41) (36), which detects tRNA and tmRNA genes in the plasmid sequences. We subsequently analyzed CRISPR/Cas systems within the curated plasmids. To detect the CRISPR arrays and associated Cas genes in each plasmid genome, we used CRISPRCasTyper (v1.8.0) (37). Furthermore, we identified the subtypes of these systems based on a comprehensive assessment of both the Cas genes and the CRISPR repeat sequences. We subsequently employed SignalP 6.0 (38) to predict the presence of signal peptides and the location of their cleavage sites in proteins. Finally, to detect the topology of membrane proteins, we used TMHMM 2.0 (39), which utilizes a hidden Markov model to identify the structural intricacies of membrane proteins. We used all of the abovementioned tools with their respective default parameters (Supplementary Table S2).

### Functional annotation

For functional annotation of coding sequences, we applied eggNOG-mapper (v2.1.12) (40) with the default parameters to the fast orthology assignments using precomputed eggNOG (v5.0.2) (41) clusters and phylogenies. The results of eggNOG annotation included a range of matching and scoring information as well as functional annotation insights from various databases, such as Gene Ontology (GO) (42), the Kyoto Encyclopedia of Genes and Genomes (KEGG) (43), the BiGG Database (44), Clusters of Orthologous Groups (COG) (45) and the Carbohydrate-Active EnZymes database (CAZy) (46) (Supplementary Table S3). Furthermore, we employed Diamond (v2.1.8.162) (47) to conduct a homology search for plasmid proteins against the Virulence Factor Database (VFDB) (48), identifying virulence factors when matches exceeded a 60% threshold of identity and a 40% threshold of coverage. We performed antibiotic resistance gene annotation based on both homology and single-nucleotide polymorphism models using reference data from the Comprehensive Antibiotic Resistance Database (CARD) (49) with the following parameters: ’*–include_loose*’ and ’*–include_nudge*’. Finally, we employed antiSMASH (v7.1.0) (50) to identify and annotate the secondary metabolite biosynthesis gene clusters within the plasmid genomes using the following parameters: ’*–asf –cc-mibig –cb-general –cb-knownclusters –cb-subclusters –pfam2go*’.

### Protein structure prediction

We predicted the sophisticated structures of the proteins and generated 3D models using advanced artificial intelligence-based modeling methods. Leveraging the large language model, ESMFold predicts full atomic-level protein structures directly from the primary sequence (51). Recognizing the potential of this advancement, we have embedded ESMFold into the web-based interface of PlasmidScope. For each predicted protein structure, we adopted the predicted local distance difference test (pLDDT) to assess the prediction quality for its residues. On the ‘Protein detail’ page, users can hover over the structure to view the pLDDT value for each residue and download the CIF file that contains pLDDT values for further application.

### Sequence alignment

To compare coding DNA sequences across a spectrum of plasmid genomes provided by users or against the plasmid genomes in PlasmidScope, we implemented BLASTP (52) to facilitate pairwise alignment of the encoded proteins. In the alignment visualizations, we showcase the alignment coverage and identity values derived from the BLAST outputs. The plasmid order in these visualizations is automatically determined to ensure optimal alignment arrangement, indicating closer genetic resemblance within each group.

### Comparative analysis

We constructed a comparative tree that illustrates the relationship among multiple plasmids using a 2-step process. First, we used Alfpy (53), an alignment-free sequence comparison method, to calculate the genomic distance (Euclidean distance based on *k*-mer = 6) between the plasmid sequences. We subsequently applied the neighbor-joining algorithm to synthesize these distances into a comparative tree, thereby providing a visual representation of the relationships among the plasmids.

### Statistical analysis

In the case study, we analyzed the length distribution of the plasmids using the ggplot2 and plyr packages in R (54,55). Based on the functional annotations obtained from PlasmidScope, we calculated the number of genes in each COG, ARG and VF category and visualized the enrichment results for the conjugative, mobilizable, and non-mobilizable plasmids using the sankeywheel package in R (https://cran.r-project.org/web/packages/sankeywheel/vignettes/sankeywheel.html). In addition, we calculated the gene numbers for the ARG categories within each plasmid and compared the distribution of cephalosporin resistance genes across the conjugative, mobilizable, and non-mobilizable plasmids using the Wilcoxon rank-sum test. The level of statistical significance was set at *P* < 0.05.

### Platform development

PlasmidScope is hosted on an Ubuntu 20.04.6 LTS server equipped with 1 TB memory and 90 TB storage. The platform’s backend functionality is supported by an in-house framework consisting of Apache, Django, PostgreSQL and Typescript+Vue3 (56,57). All online data visualizations are implemented using Oviz (58). We also provide detailed tutorials on the platform to facilitate user navigation and utilization.

## Results

### Comprehensive annotated plasmid database in PlasmidScope

**Figure 1.**
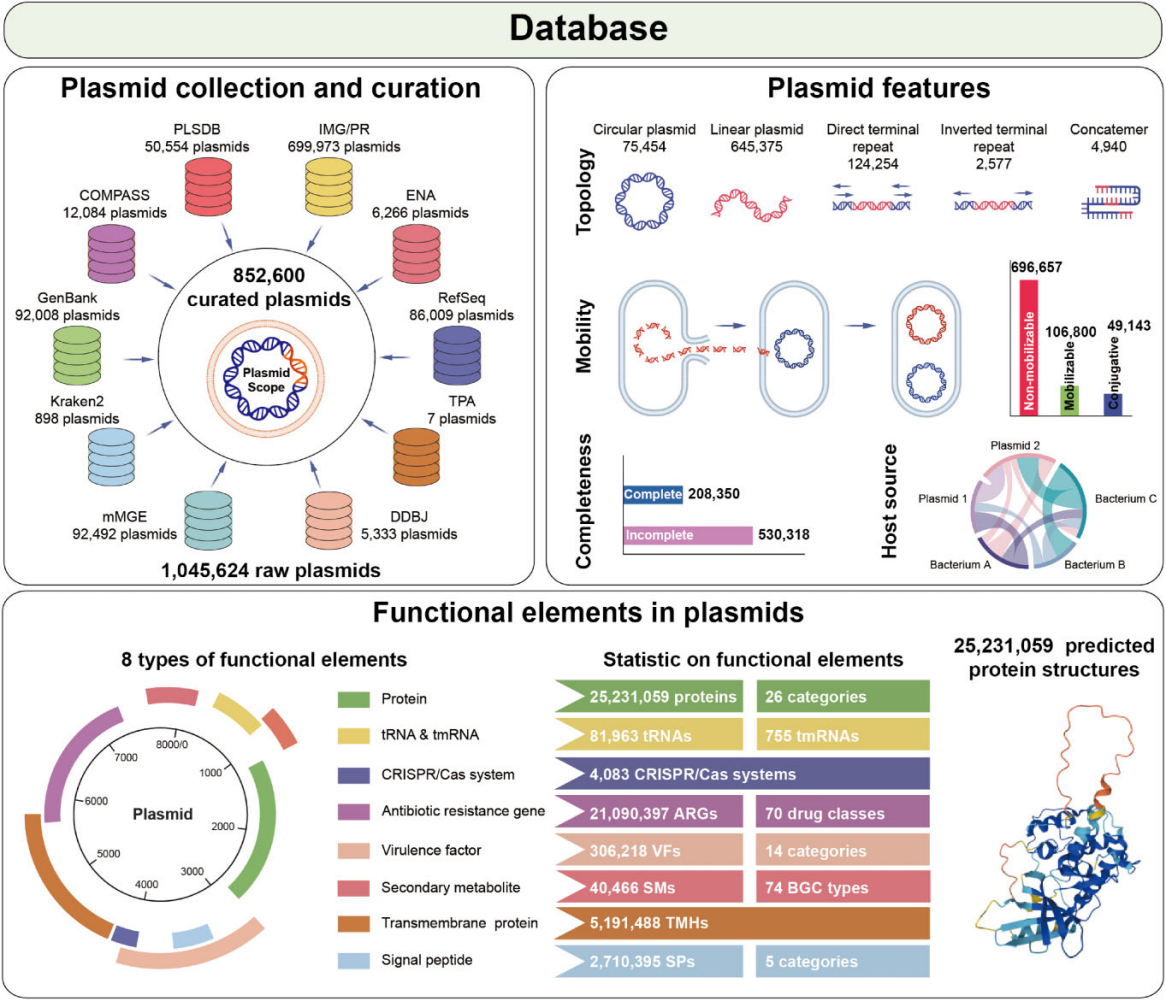
Overview of the plasmid database in PlasmidScope.PlasmidScope contains a comprehensive database of 852,600 plasmids curated from 10 repositories along with standardized and comprehensive annotations, including feature consolidation, genetic feature annotation, gene function annotation, and protein structure prediction.

PlasmidScope contains a comprehensive database of 852 600 plasmids curated from 10 repositories (Figure 1), including 8 repositories that isolate plasmids from single microorganisms and 2 repositories that incorporate metagenomic and metatranscriptomic data (Supplementary Table S1). The plasmid repositories, PLSDB (20), COMPASS (21), GenBank (23), RefSeq (24), ENA (27), DDBJ (28), Kraken2 (29) and TPA (30), contain 110,021 plasmids isolated from single microorganisms, constituting 12.90% of the PlasmidScope database. Meanwhile, the plasmid repositories that incorporate metagenomic and metatranscriptomic data, IMG/PR (25) and mMGE (26), adopt homologous sequence alignment or deep learning to capture 742,579 plasmids from assembled metagenomes and metatranscriptomes, constituting 87.10% of the PlasmidScope database. To broaden the applications of these plasmids, PlasmidScope systematically encompasses each curated plasmid with standardized and comprehensive annotations that can be summarized into 3 main categories: feature, genetic feature and gene feature.

PlasmidScope collects and consolidates six features of the curated plasmids: genomic length, GC content, genome completeness, topological structure, mobility and host source (Figure 1). The genomic length of the plasmids ranges from 200 to 11 850 240 bp, while their GC content ranges from 11.73% to 87.48%. Among the curated plasmids, 208 350 have complete genomes. Regarding topological structure, PlasmidScope categorizes plasmids as circular plasmids (75 454 sequences), linear plasmids (645 375 sequences), direct terminal repeats (124 254 sequences), inverted terminal repeats (2577 sequences), or concatemers (4940 sequences). Meanwhile, 696 657 (81.71%) plasmids are non-mobilizable plasmids, followed by 106 800 (12.53%) mobilizable and 49 143 (5.76%) conjugative plasmids. In addition, the host distributions of 360 583 (42.29%) plasmids are available in PlasmidScope, which are mainly abundant in Enterobacterales (28.63%), Bacillales (9.24%) and Bacteroidales (7.92%). These precisely integrated features assist users in exploring the basic characteristics of plasmids and selecting appropriate and efficient plasmids for further use, such as bioengineering vector selection.

PlasmidScope provides six genetic features of the curated plasmids: gene, tRNA, tmRNA, signal peptide, transmembrane protein and CRISPR/Cas system (Figure 1, Supplementary Table S4). By screening the plasmids through the standardized bioinformatic pipeline, we identified 25 231 059 genes, 81 963 tRNAs and 755 tmRNAs. In addition, PlasmidScope includes 2,710,395 signal peptides that can be categorized into five classes: Sec signal peptide (SP, 71.43%), Lipoprotein signal peptide (LIPO, 22.00%), Tat signal peptide (TAT, 5.06%), Tat lipoprotein signal peptide (TATLIPO, 0.50%), and Pilin signal peptide (PILIN, 1.01%). PlasmidScope provides detailed structural information for 5 191 488 identified transmembrane proteins, including the number, order and amino acid sequences of the transmembrane segments, thereby facilitating the exploration of the effects of structures on the transport of specific substances. Furthermore, PlasmidScope offers detailed information on 4083 predicted CRISPR/Cas systems, including types, subtypes, Cas genes and consensus repeat sequences. The exhaustive annotated genetic features enable users to investigate the structures of genetic elements, facilitating related structure design and optimization.

PlasmidScope contains comprehensive functional annotations and advanced translated protein structural prediction for each predicted gene (Figure 1). To investigate the functions of plasmids, PlasmidScope performs functional annotations for the 25,231,059 predicted genes with 9 databases, including GO (42), KEGG (43), BiGG (44), COG (45), CAZy (46), VFDB (48), CARD (49), Pfam (59) and MIBiG (60). In addition, PlasmidScope provides the specific functional category information underlying each database, such as COG category, GO class and KEGG pathway. To provide an in-depth view into the natural diversity of proteins, PlasmidScope predicts the protein structures of genes with ESMFold and produces high-resolution visualizations of protein structures that show atomic-level structures. Notably, PlasmidScope supports both online viewing adjustment of protein structures and the downloading of protein structure files. The comprehensive functional annotations and advanced protein structure predictions can accelerate the discovery of horizontal-transferring functional genes and the detection of protein structures.

### Integrated online analytical modules in PlasmidScope

**Figure 2.**
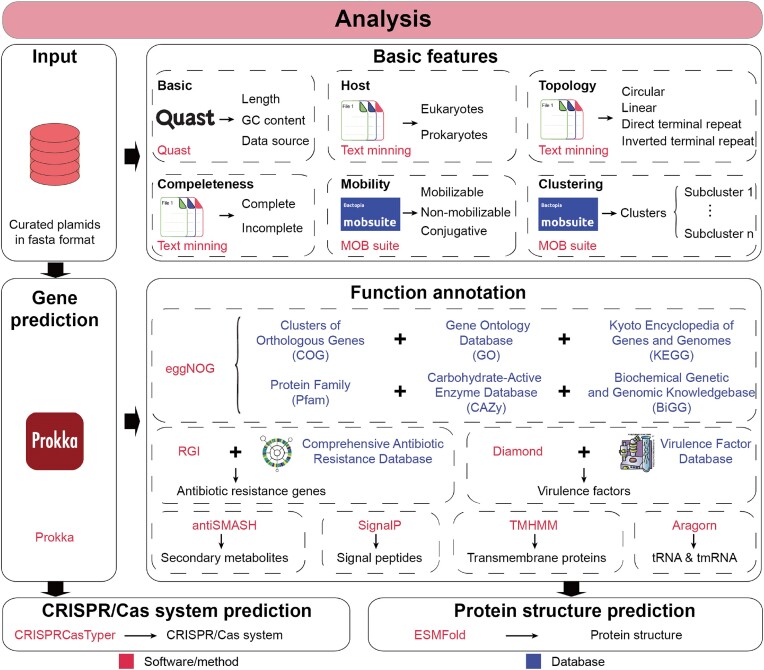
Overview of the analytical procedure of PlasmidScope. PPlasmidScope uses bioinformatics tools and eight functional databases to perform standardized annotations for each plasmid sequence. In addition, PlasmidScope contains six analytical modules and two analytical pipelines, enabling users to perform online plasmid analysis.

PlasmidScope offers online analytical modules that allow users to upload their custom plasmids, perform functional and structural annotations, and make comparisons with plasmids in the database. Users can upload single or multiple plasmid sequences and choose from 6 analytical modules: open reading frame prediction and protein annotation, tRNA and tmRNA prediction, ARG and VF annotation, transmembrane protein annotation, sequence alignment, and comparative analysis—all of which utilize the advanced tools detailed in the Materials and methods section (Figure 2). Furthermore, PlasmidScope integrates these six modules into two analytical pipelines according to their application field: plasmid annotation and plasmid comparison. The plasmid annotation pipeline includes open reading frame prediction and protein annotation, tRNA and tmRNA prediction, ARG and VF annotation, and transmembrane protein annotation. Meanwhile, the plasmid comparison pipeline includes sequence alignment and comparative analysis. Users can perform either single or multiple analyses based on their specific research needs and objectives. After the analysis is complete, users can check and download the resulting documents and visualizations, supporting further plasmid research.

### Informative and interactive visualizations in PlasmidScope

**Figure 3.**
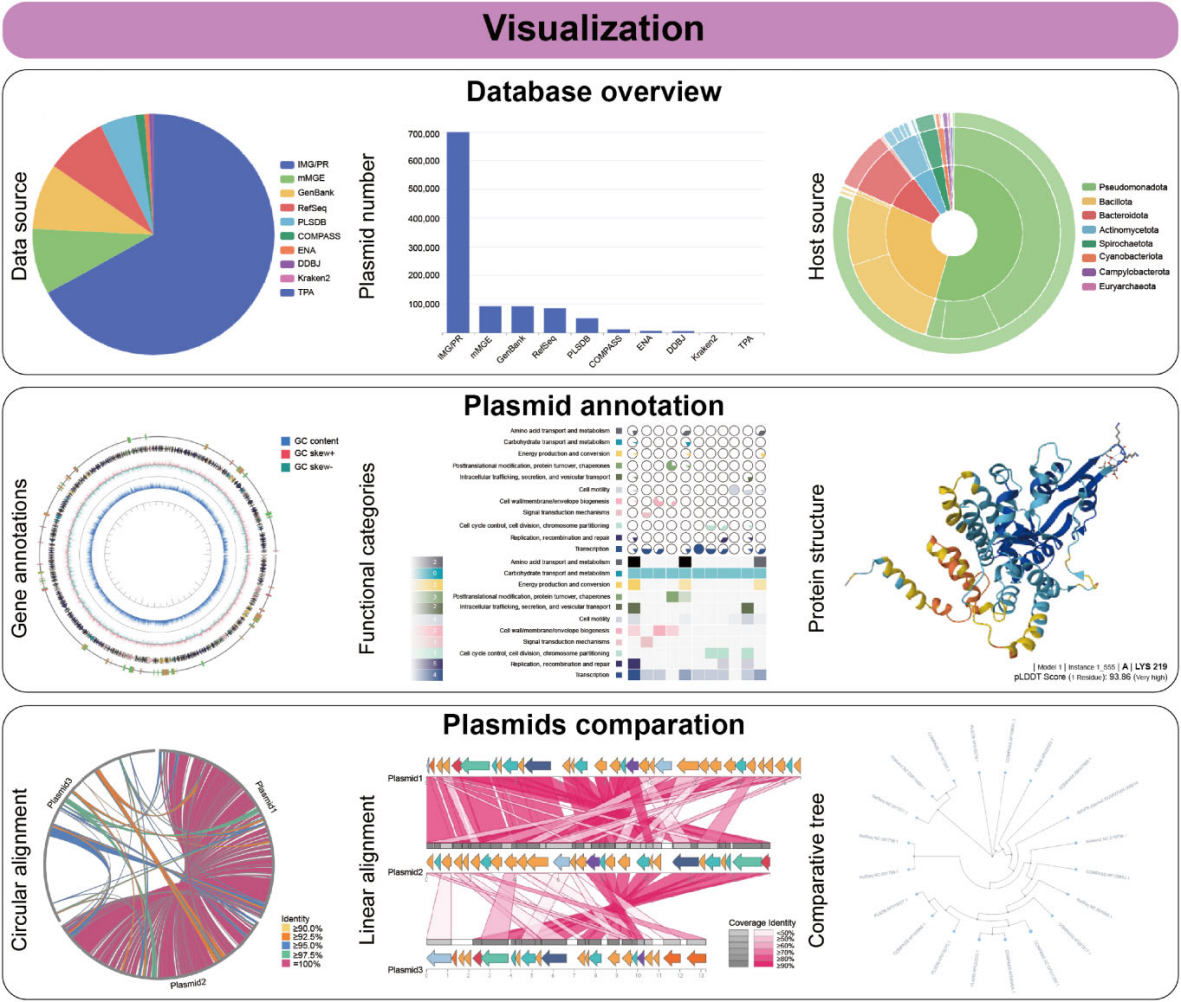
Overview of interactive visualizations in PlasmidScope. PlasmidScope generates interactive visualizations of its curated database along with comprehensive annotations. In addition, analysis results and visualizations generated from the online analytical modules or pipelines of PlasmidScope are downloadable.

PlasmidScope features a user-friendly interface that enables users to seamlessly query, visualize, analyze, and download information. PlasmidScope has 7 menus: ‘Home’, ‘Database’, ‘Analysis’, ‘Workspace’, ‘Download’, ‘Tutorial’ and ‘Contact Us’. The homepage provides a basic summary of PlasmidScope, three key feature plots, and version updates. The ‘Database’ menu offers a concise summary page (Figure 3), 11 detailed annotation pages, and a querying page. Each of the 11 annotation pages presents key features from various perspectives, such as plasmid feature, host source, cluster information, and protein annotation, along with a search box in the upper-right corner. In addition, some annotation pages contain ‘Detail’ and ‘Download’ buttons, allowing users to view detailed annotation results and download related sequences, respectively. As described above, the ‘Analysis’ menu comprises two pages: ‘Plasmid Annotation’ and ‘Plasmid Comparison’, which contain four and two analytical modules, respectively. The ‘Workspace’ menu displays the status of submitted tasks. Users can click the ‘View Result’ button for each task to access and download their custom analysis and visualization results, which can be easily integrated into academic publications (Figure 3). The ‘Download’ menu provides a list of available files for download. Finally, the ‘Tutorial’ menu offers user detailed instructions on how to use PlasmidScope, and the ‘Contact Us’ menu facilitates convenient communication with the support team.

### Case study: plasmids as reservoirs of antibiotic-resistance genes in *Klebsiella pneumoniae*

As an opportunistic pathogen, *Klebsiella pneumoniae* can cause pneumonia, urinary tract infection, bacteremia, and other infections when the host’s immune function is compromised (61). In recent years, the overuse of antibiotics has led to the emergence and global spread of hypervirulent *K. pneumoniae*, which has been reported in 43 countries and exhibits resistance to third-generation cephalosporins, posing a significant threat to public health (https://www.who.int/emergencies/disease-outbreak-news/item/2024-DON527). As one of the main vehicles for horizontal gene transfer, plasmids offer opportunities to deepen our understanding on the emergence of *K. pneumoniae* multidrug resistance.

**Figure 4.**
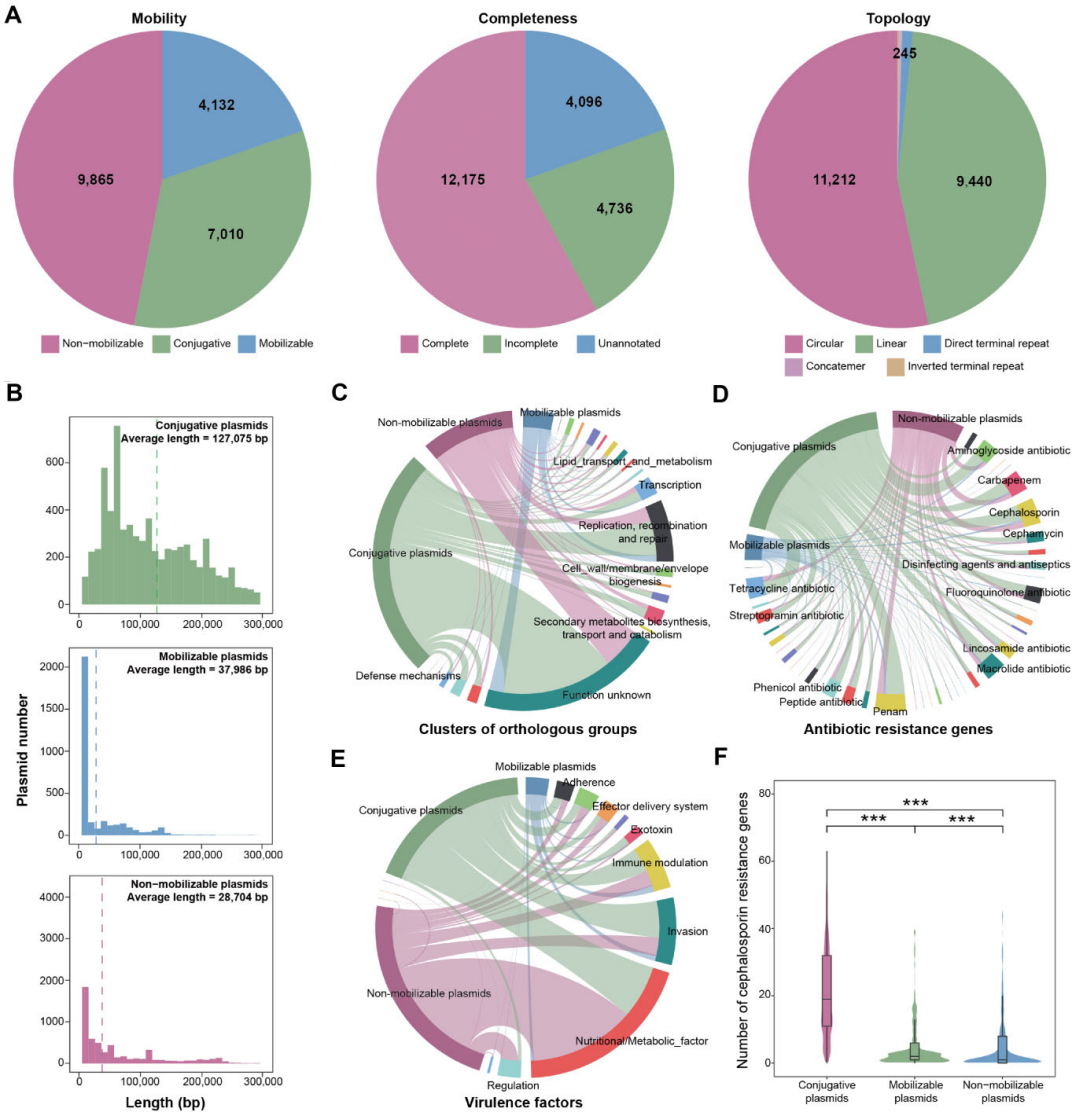
Case study: using PlasmidScope to investigate the antibiotic resistance genes in plasmids from *Klebsiella pneumoniae*. (**A**) Mobility, completeness and topological features in the 21 007 plasmids from *K. pneumoniae*. (**B**) Length distributions of the conjugative, mobilizable and non-mobilizable plasmids. (C–E) Gene enrichment results based on the Clusters of Orthologous Groups database (**C**) Comprehensive Antibiotic Resistance Database (**D**) and Virulence Factor Database. (**E**) Dashed lines indicate the average length of the plasmids. (**F**) Cephalosporin resistance gene numbers across the conjugative, mobilizable and non-mobilizable plasmids. *** *P* < 0.001, Wilcoxon rank-sum test.

Accordingly, we used PlasmidScope to analyze the distribution and characteristics of ARGs in plasmids from *K. pneumoniae*. By accessing the plasmids under the ‘Host list’ page in PlasmidScope, we collected 21 007 plasmids from *K. pneumoniae*, including 7010 conjugative, 4132 mobilizable and 9865 non-mobilizable plasmids (Figure 4A). Using the feature annotations provided by PlasmidScope, we further identified 12 175 complete plasmids and 11 212 circular plasmids (Figure 4A). Upon analyzing plasmid length distributions, we found that the conjugative plasmids had the longest genome (127 075.43 ± 83 573.62 bp), followed by the non-mobilizable (37 986.33 ± 83 788.18 bp) and mobilizable plasmids (28 704.41 ± 42 249.88 bp) (Figure 4B). These plasmids harbored 1 579 934 predicted genes, including 1 234 887 ARGs and 15 575 VFs (Figure 4C–E). The conjugative, mobilizable and non-mobilizable plasmids contained substantial COGs particularly enriched in the categories of ‘replication, recombination and repair’ (17.00%, 22.21% and 17.40%, respectively), ‘transcription’ (4.46%, 5.59% and 5.72%, respectively), and ‘inorganic ion transport and metabolism’ (4.03%, 2.83% and 5.33%, respectively) (Figure 4C). ARG enrichment indicated that all three types of plasmids mainly had resistance against ‘penam’ (11.50%) and ‘cephalosporin’ (9.44%) (Figure 4D). VF enrichment analysis revealed that the ‘invasion’ function was abundant in the conjugative and mobilizable plasmids (24.47% and 28.82%, respectively), but less prevalent in the non-mobilizable plasmids (9.99%) (Figure 4E). Furthermore, the conjugative and mobilizable plasmids carried significantly more antibiotic resistance genes against cephalosporin than the non-mobilizable plasmids (*P* < 0.001, Figure 4F), offering insights into the acquisition of multidrug-resistance in *K. pneumoniae*. Thus, these findings demonstrate the utility of PlasmidScope as a convenient tool in the face of the antimicrobial resistance crisis.

## Discussion

To our knowledge, PlasmidScope is the largest and most comprehensive plasmid database that integrates built-in analytical tools and interactive visualization to help researchers decipher the physiological and genetic characteristics of plasmids across different microbial hosts. PlasmidScope has the following key features: (i) an extensive collection of over 1 million plasmids with standardized and comprehensive annotations; (ii) online analytical modules that support customized plasmid annotation and plasmid comparison; (iii) interactive visualization of the curated database, detailed annotations, and customizable analysis results; (iv) downloadable support for all resources. PlasmidScope is freely available to the public and can be accessed without registration.

In addition to its extensive collection of plasmids, PlasmidScope has several key advantages. First, PlasmidScope is closely integrated with existing databases, retaining the IDs and background information of plasmids in other databases, which enables connections and cross-comparison among databases. Second, PlasmidScope enables comprehensive, accurate, and downloadable annotations, which are essential for investigating plasmid genetic features. Third, PlasmidScope contains integrated online analytical modules to support plasmid annotation, plasmid comparison, and visualization. Fourth, PlasmidScope offers informative and interactive visualizations for the curated database, related annotations, and online analysis results. Finally, PlasmidScope integrates ESMFold to help users predict and view protein structures, which is not possible with other plasmid databases. Hence, PlasmidScope is a powerful platform that supports plasmid genome research and genome-scale genetic element analysis.

By serving the scientific community, PlasmidScope can become a centralized platform for plasmid research. We will also continually update and enhance PlasmidScope. First, we plan to expand PlasmidScope by incorporating additional plasmid databases to broaden its applicability and facilitate cross-referencing of information across studies. Second, we plan to embed more deep-learning models and bioinformatic modules, such as completement assessment and host prediction, to support online analysis and data mining. Third, we will continuously optimize PlasmidScope’s framework and interface based on user feedback, enhancing its usability. Last, we aim to establish a secure and reliable system to facilitate big data exchange and support the archiving of plasmid data for users.

In conclusion, PlasmidScope is a valuable resource with practical tools that will contribute to productive research endeavors and empower researchers to delve deeper into the intricacies of plasmids.

## Supplementary Material

gkae930_Supplemental_File

## Data Availability

All data are freely available at https://plasmid.deepomics.org/.
