# Supplementary material for: PlasmidScope: a comprehensive plasmid database with rich annotations and online analytical tools
Source: Nucleic Acids Res. 2024 Oct 23;53(D1):D179–88. doi: 10.1093/nar/gkae930 (PMC11701673; doi:10.1093/nar/gkae930)
Supplement: gkae930_Supplemental_File [file gkae930_supplemental_file.pdf]

PlasmidScope: A comprehensive plasmid database with rich annotations and online analytical tools

Yinhu Li[†], Xikang Feng[†], Xuhua Chen[†], Shuo Yang, Zicheng Zhao, Yu Chen[*],and Shuai Cheng Li[*]

October 2, 2024

List of Tables

S1 Details of plasmid sequence collection 2
S2 Tools and parameters used for plasmid analysis 2
S3 Databases used for functional annotation 3
S4 Genomic elements and functional genes stored in PlasmidScope 3

List of Figures

S1 Plasmid curation process. 4

S1 Supplementary Tables

Supplementary Table S1: Details of plasmid sequence collection

Dataset	# of sequences	Data source	Website	Publications
RefSeq	86,009	NCBI	https://www.ncbi.nlm.nih.gov/refseq/	[1]
Genbank	923,008	NCBI	https://www.ncbi.nlm.nih.gov/genbank/	[2]
ENA	6,266	EMBL	https://www.ebi.ac.uk/ena/browser/home	[3]
DDBJ	5,333	DDBJ	https://www.ddbj.nig.ac.jp/index-e.html	[4]
Kraken2	898	NCBI	https://benlangmead.github.io/aws-indexes/k2	[5]
TPA	7	NCBI	https://www.ncbi.nlm.nih.gov/genbank/tpa/	[6]
PLSDB	50,554	NCBI	https://ccb-microbe.cs.uni-saarland.de/plsdb/	[7]
COMPASS	12,084	NCBI	https://github.com/itsmeludo/COMPASS	[8]
IMG/PR	699,973	IMG datasets	https://img.jgi.doe.gov/pr	[9]
mMGE	92,492	Human metagenome	https://mai.fudan.edu.cn/mgedb/client/	[10]

Supplementary Table S2: Tools and parameters used for plasmid analysis

Assignment	Tool	Parameter
Deduplication	MMseqs2	–cov mode 0 -c 1.0 –min-seq-id 1.0
Mobility prediction	MOB-suite	Defaults
Clustering	MOB-suite	Defaults
Gene prediction	Prokka	Defaults
Genetic elements annotation	CRISPRCasTyper	Defaults
	SignalP	Defaults
	TMHMM	Defaults
Functional annotation	Diamond	–evalue 1e-5 –id 60 –subject-cover 40
	RGI	–include_loose –include_nudge
	antiSMASH	-asf –cc-mibig –cb-general –cb-knownclusters –cb-subclusters –pfam2go
Protein structure prediction	ESMFold	Defaults
Phylogenetic analysis	Alfpy	-word_size 6 -distance euclid_norm -vector counts

Supplementary Table S3: Databases used for functional annotation

Database	Full name	Website	Publication
COG	Database of Clusters of Orthologous Genes	https://ccb-microbe.cs.uni-saarland.de/plsdb/	[11]
GO	Gene Ontology	https://www.geneontology.org/	[12]
KEGG	Kyoto Encyclopedia of Genes and Genomes	https://www.genome.jp/kegg/	[13]
Pfam	Protein Families Database	http://pfam.xfam.org/	[14]
CAZy	Carbohydrate-Active enZYmes Database	http://www.cazy.org/	[15]
BiGG	Biochemical Genetic and Genomic knowledgebase	http://bigg.ucsd.edu/	[16]
CARD	The Comprehensive Antibiotic Resistance Database	https://card.mcmaster.ca/	[17]
VFDB	Virulence Factor Database	http://www.mgc.ac.cn/VFs/	[18]
MIBiG	Minimum Information about a Biosynthetic Gene cluster	https://mibig.secondarymetabolites.org/	[19]

Supplementary Table S4: Genomic elements and functional genes stored in PlasmidScope

Database	tRNAs & tmRNAs	CRISPR/Cas systems	Signal peptides	Transmembrane protein	Virulence factor	Antibiotic resistance gene	Secondary metabolism region
RefSeq	18,939	2,079	720,753	1,311,177	106,425	5,337,195	12,188
GenBank	17,241	1,967	706,270	1,313,227	112,680	5,306,079	12,424
ENA	1,206	88	72,047	76,281	4,997	312,019	452
DDBJ	1,275	317	45,449	83,827	6,831	374,767	666
Kraken2	179	13	5,304	10,749	816	42,956	82
TPA	1	0	38	129	13	583	2
PLSDB	14,385	1,482	570,217	1,345,493	94,889	4,507,567	9,010
COMPASS	2,685	489	130,636	465,289	21,045	1,085,253	1,913
IMG/PR	53,599	3,653	1,921,273	3,221,059	165,704	14,076,411	27,000
mMGE	8,661	382	96,630	408,801	20,395	873,294	1,271

S2 Supplementary Figures

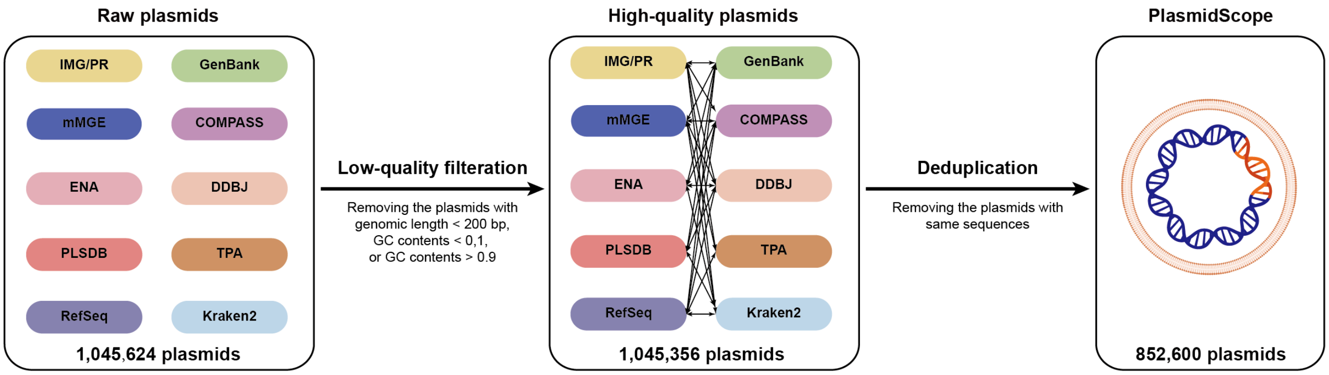

Supplementary Figure S1: **Plasmid curation process.**

References

[1] O'Leary, N.A., Wright, M.W., Brister, J.R., Ciufo, S., Haddad, D., McVeigh, R., Rajput, B., Robbertse, B., Smith-White, B., Ako-Adjei, D. et al. (2016) Reference sequence (RefSeq) database at NCBI: current status, taxonomic expansion, and functional annotation. *Nucleic Acids Res*, **44**, D733-745.

[2] Benson, D.A., Cavanaugh, M., Clark, K., Karsch-Mizrachi, I., Ostell, J., Pruitt, K.D. and Sayers, E.W. (2018) GenBank. *Nucleic Acids Res*, **46**, D41-D47.

[3] Kulikova, T., Akhtar, R., Aldebert, P., Althorpe, N., Andersson, M., Baldwin, A., Bates, K., Bhattacharyya, S., Bower, L., Browne, P. et al. (2007) EMBL Nucleotide Sequence Database in 2006. *Nucleic Acids Res*, **35**, D16-20.

[4] Ogasawara, O., Kodama, Y., Mashima, J., Kosuge, T. and Fujisawa, T. (2020) DDBJ Database updates and computational infrastructure enhancement. *Nucleic Acids Res*, **48**, D45-D50.

[5] Lu, J., Rincon, N., Wood, D.E., Breitwieser, F.P., Pockrandt, C., Langmead, B., Salzberg, S.L. and Steinegger, M. (2022) Metagenome analysis using the Kraken software suite *Nat Protoc*, **17**, 2815-2839.

[6] Cochrane, G., Bates, K., Apweiler, R., Tateno, Y., Mashima, J., Kosuge, T., Mizrachi, I.K., Schafer, S. and Fetchko, M. (2006) Evidence standards in experimental and inferential INSDC Third Party Annotation data. *OMICS*, **10**, 105-113.

[7] Schmartz, G.P., Hartung, A., Hirsch, P., Kern, F., Fehlmann, T., Muller, R. and Keller, A. (2022) PLSDB: advancing a comprehensive database of bacterial plasmids. *Nucleic Acids Res*, **50**, D273-D278.

[8] Douarre, P.E., Mallet, L., Radomski, N., Felten, A. and Mistou, M.Y. (2020) Analysis of COMPASS, a New Comprehensive Plasmid Database Revealed Prevalence of Multireplicon and Extensive Diversity of IncF Plasmids. *Front Microbiol*, **11**, 483.

[9] Camargo, A.P., Call, L., Roux, S., Nayfach, S., Huntemann, M., Palaniappan, K., Ratner, A., Chu, K., Mukherjeep, S., Reddy, T.B.K. et al. (2024) IMG/PR: a database of plasmids from genomes and metagenomes with rich annotations and metadata. *Nucleic Acids Res*, **52**, D164-D173.

[10] Lai, S., Jia, L., Subramanian, B., Pan, S., Zhang, J., Dong, Y., Chen, W.H. and Zhao, X.M. (2021) mMGE: a database for human metagenomic extrachromosomal mobile genetic elements. *Nucleic Acids Res*, **49**, D783-D791.

[11] Galperin, M.Y., Wolf, Y.I., Makarova, K.S., Vera Alvarez, R., Landsman, D. and Koonin, E.V. (2021) COG database update: focus on microbial diversity, model organisms, and widespread pathogens. *Nucleic Acids Res*, **49**, D274-D281.

[12] The Gene Ontology, C. (2017) Expansion of the Gene Ontology knowledgebase and resources. *Nucleic Acids Res*, **45**, D331-D338.

[13] Kanehisa, M., Furumichi, M., Tanabe, M., Sato, Y. and Morishima, K. (2017) KEGG: new perspectives on genomes, pathways, diseases and drugs. *Nucleic Acids Res*, **45**, D353-D361.

[14] Mistry, J., Chuguransky, S., Williams, L., Qureshi, M., Salazar, G.A., Sonnhammer, E.L.L., Tosatto, S.C.E., Paladin, L., Raj, S., Richardson, L.J. et al. (2021) Pfam: The protein families database in 2021. *Nucleic Acids Res*, **49**, D412-D419.

[15] Drula, E., Garron, M.L., Dogan, S., Lombard, V., Henrissat, B. and Terrapon, N. (2022) The carbohydrate-active enzyme database: functions and literature. *Nucleic Acids Res*, **50**, D571-D577.

[16] Schellenberger, J., Park, J.O., Conrad, T.M. and Palsson, B.O. (2010) BiGG: a Biochemical Genetic and Genomic knowledgebase of large scale metabolic reconstructions. *BMC Bioinformatics*, **11**, 213.

[17] Alcock, B.P., Huynh, W., Chalil, R., Smith, K.W., Raphenya, A.R., Wlodarski, M.A., Edalatmand, A., Petkau, A., Syed, S.A., Tsang, K.K. et al. (2023) CARD 2023: expanded curation, support for machine learning, and resistome prediction at the Comprehensive Antibiotic Resistance Database. *Nucleic Acids Res*, **51**, D690-D699.

[18] Liu, B., Zheng, D., Zhou, S., Chen, L. and Yang, J. (2022) VFDB 2022: a general classification scheme for bacterial virulence factors. *Nucleic Acids Res*, **50**, D912-D917.

[19] Terlouw, B.R., Blin, K., Navarro-Munoz, J.C., Avalon, N.E., Chevrette, M.G., Egbert, S., Lee, S., Meijer, D., Recchia, M.J.J., Reitz, Z.L. et al. (2023) MIBiG 3.0: a community-driven effort to annotate experimentally validated biosynthetic gene clusters. *Nucleic Acids Res*, **51**, D603-D610.
